# Metabolic Dysfunction-associated Fatty Liver Disease: An Urgent Call for Global Action

**DOI:** 10.17925/EE.2023.20.1.1

**Published:** 2023-11-08

**Authors:** Cornelius J Fernandez, Lakshmi Nagendra, Joseph M Pappachan

**Affiliations:** 1. Department of Endocrinology & Metabolism, Pilgrim Hospital, United Lincolnshire Hospitals NHS Trust, Boston, UK; 2. Department of Endocrinology, JSS Medical College, JSS Academy of Higher Education and Research, Mysore, India; 3. Department of Endocrinology & Metabolism, Lancashire Teaching Hospitals NHS Trust, Preston, UK; 4. Faculty of Science, Manchester Metropolitan University, Manchester, UK; 5. Faculty of Biology, Medicine and Health, The University of Manchester, Manchester, UK

**Keywords:** Fatty liver, metabolic-associated fatty liver disease (MAFLD), metabolic syndrome, non-alcoholic fatty liver disease, obesity, obstructive sleep apnoea, polycystic ovary syndrome, type 2 diabetes mellitus

## Abstract

There has been an exponential increase in the global prevalence of fatty liver disease in recent years in association with the obesity pandemic worldwide. 'Metabolic dysfunction-associated fatty liver disease', the new terminology adopted by an international panel of experts in 2020 to largely replace the old term 'non-alcoholic fatty liver disease', has now been accepted by most hepatologists and diabetologists across the globe. The term metabolic dysfunction-associated fatty liver disease was created to better reflect the metabolicand liver-specific manifestations and complications of fatty liver disease. It is important to disseminate our current understanding of this enigmatic disease among the global scientific fraternity. Recent publications, including articles from the latest issue of *Endocrinology & Metabolism Clinics of North America*, are attempting to fill this knowledge gap.

There has been an exponential increase in the global prevalence of obesity over the past few decades because of adverse lifestyle choices, such as physical inactivity and overconsumption of macronutrients. The obesity pandemic has contributed to more than 50 different disorders related to excess body weight, which has substantially increased morbidity and mortality worldwide.^1^ Metabolic syndrome (MetS) is one of the most common obesity-related morbidities.^2^ As the name indicates, this condition results in the metabolic dysfunction of various body tissues, including the liver. This dysfunction often leads to steatosis (fat deposition) in the liver, resulting in the development of fatty liver disease. This editorial aims to disseminate the understanding regarding metabolic dysfunction-associated fatty liver disease based on the current evidence, with a focus on a recent issue of *Endocrinology and Metabolism Clinics of North America*.

The term ‘non-alcoholic fatty liver disease’ (NAFLD) was first coined in the 1980s by Ludwig et al. to describe the pathobiological aspects of liver steatosis.^3^ Over the years, a better understanding of the disease process has resulted in a change in nomenclature to a more appropriate term: metabolic (dysfunction)-associated fatty liver disease (MAFLD). This term was coined in 2020 by an international consensus panel including experts from 22 countries.^4^ As per this consensus, MAFLD is diagnosed when an individual presents with hepatic steatosis of ≥5%, coupled with one of the following three different phenotypes: type 2 diabetes mellitus in individuals who may or may not have a healthy body mass index (BMI); overweight or obesity based on ethnic-specific BMI criteria; healthy weight by ethnic-specific BMI criteria but with abnormalities in at least two out of the seven risk factors of MetS ( waist circumference, blood pressure, triglycerides, high-density lipoprotein cholesterol, prediabetes, homeostasis model assessment of insulin resistance score and high-sensitivity C-reactive protein).^4^

In contrast to NAFLD, the presence of a metabolic abnormality is necessary for the diagnosis of MAFLD. However, excluding alcohol intake or other chronic liver disorders, including viral hepatitis, is not required for diagnosing MAFLD (*Figure 1*).^5^ The pathogenic process that causes MAFLD is now better understood and is believed to result from a state of systemic metabolic malfunction. The increased prevalence of MAFLD makes it very likely that it will coexist with other chronic liver diseases.^4^ Hence, with the new diagnostic criteria for MAFLD, dual pathologies for MAFLD, such as viral hepatitis and autoimmune hepatitis, can be considered as they can be associated with higher rates of advanced liver fibrosis. The new diagnostic criteria for MAFLD also allow to diagnose lean MAFLD as a distinct clinical entity that carries similar or even higher complication rates compared with overweight or obese MAFLD and can be defined as patients who are of normal or lean weight with ≥2 metabolic dysregulation risk factors.^5,6^ A recent study has shown that an MAFLD diagnosis is better correlated with advanced liver fibrosis and noninvasive indicators of fatty infiltration.^7^ The fact that metabolic dysregulation pathways are now recognised as having a great impact on liver disease shows that the differences between the MAFLD diagnostic criteria and the NAFLD exclusion criteria require further evaluation.

**Figure 1:**
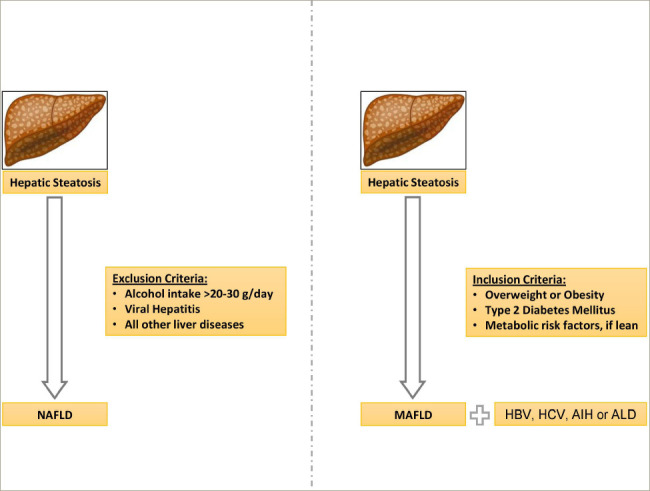
A schematic diagram outlining the differences in the diagnostic criteria between non-alcoholic fatty liver disease and metabolic-associated fatty liver disease

*AIH = autoimmune hepatitis; ALD = alcoholic fatty liver disease; HBV = hepatitis B virus; HCV = hepatitis C virus; MAFLD = metabolic-associated fatty liver disease; NAFLD = non-alcoholic fatty liver disease.*

Although many studies on NAFLD/MAFLD have been published over the past few decades, knowledge gaps in field remain. To compile the latest evidence in this area, Fouda and Pappachan edited a journal issue entitled “Metabolic-associated fatty liver disease“, which compiled 12 review articles authored by global experts in the field from nine different countries.^8^ The articles of this journal issue discuss various aspects of MAFLD, providing the latest clinical updates on the disease.

There is a bidirectional relationship between MetS and MAFLD: MetS can predispose to and exacerbate MAFLD; therefore, MAFLD can be considered the hepatic manifestation of MetS.^9^ Conversely, MAFLD can predispose to various components of MetS.^10^ In recent years, the prevalence of MAFLD has increased several-fold, in parallel with the epidemics of diabetes mellitus and obesity. MAFLD now affects 20–45% of the global population.^11,12^ The prevalence of MAFLD can be as high as 80–90% in adults who are obese, 30–50% in adults with diabetes mellitus, 70% in adults with obesity and diabetes mellitus and up to 90% in those with hyperlipidemia.^13^ This alarming rise in the prevalence of these metabolic disorders is expected to cause a substantial increase in liver-related morbidity, healthcare costs and mortality.^14^

The rise in the prevalence of childhood obesity has caused an exponential increase in the prevalence of MAFLD in children and adolescents. According to the most recent data, the prevalence of childhood obesity-related MAFLD is 52.1%, 39.7% and 23.0% in Asia, South America and Europe, respectively.^15^ The estimated global prevalence of the disease among children is 5–10%.^16^ The disease prevalence can be as high as 40–70% in children who are obese; consequently, the rate of liver-related morbidity and mortality is expected to increase in the coming years.^15^ The pharmacotherapeutic options for paediatric MAFLD cases are limited compared with the options for adults with MAFLD, as most of the agents with potentially disease-modifying properties are still being investigated in clinical trials.^17^

MAFLD may slowly progress from hepatic steatosis to non-alcoholic steatohepatitis (NASH), fibrosis, cirrhosis and, at times, hepatocellular carcinoma (HCC).^13^ MAFLD is now the most common cause of chronic liver disease and a leading cause of cirrhosis and HCC worldwide.^11^ Nearly 10% of patients with MAFLD progress to the stage of NASH, and nearly 41% of patients with NASH progress to fibrosis; the latter occurrs in around 7–14 years, depending on the stage of hepatitis.^18^ As such, NASH and fibrosis are risk factors for HCC in some individuals, even in the absence of cirrhosis. In fact, 50% of cases of MAFLD-related HCC occur in patients without histological evidence of cirrhosis.^19^

The pathogenesis of MAFLD and its progression is complex and incompletely understood. According to the ‘two-hit’ hypothesis, it is triggered by the insulin resistance-mediated hepatic accumulation of lipids (free fatty acids and unesterified free cholesterol), leading to steatosis.^18^ Steatosis is followed by increased oxidation of fatty acids, leading to the production of reactive oxygen species and toxic lipid species (ceramides and diacylglycerides). This production then leads to lipotoxicity and inflammation, mediated by DNA damage, mitochondrial damage, lysosomal dysfunction, endoplasmic reticulum stress and the release of proinflammatory cytokines. The recruitment of Kupffer cells and the activation of hepatic stellate cells result in fibrosis, cirrhosis and possibly HCC.^18^

However, the ‘two-hit’ hypothesis is too simplistic to explain how the complex interaction between genetic susceptibility and environmental factors, including diet, lifestyle, physical fitness, sleep sufficiency and stress, can lead to the progression of MAFLD.^20^ Hence, the ‘multi-parallel hit’ or ‘multi-hit’ hypothesis, whereby various mechanisms, including genetic polymorphisms, epigenetics, bile acids, gut microbiota alterations, sarcopenia, hypogonadism and obstructive sleep apnoea (OSA), are thought to act synergistically in MAFLD pathogenesis, was proposed.^20^ This hypothesis may also explain the complexities behind fibrogenesis and oncogenesis in patients with MAFLD.^21^ The susceptibility to MAFLD is increased by several genes, including *PNPLA3, TM6SF2, MBOAT7, HSD17B13* and *GCKR-P446L*, and transcription factors, including sterol regulatory element binding protein 2 (SREBP-2), farnesoid X receptor (FXR), and liver X receptor (LXR), which are involved in lipid metabolism.^19^

A high-calorie diet containing either excessive amounts of fats or carbohydrates is the main environmental factor involved in the pathogenesis of MAFLD.^22^ A sedentary lifestyle is associated with an increased prevalence of MAFLD and poor hepatic outcomes.^23^ Acquired hepatotropic virus infections can increase the risk of developing MAFLD and of disease progression.^22^ Conversely, steatosis can impair the hepatic microbial clearance and increase the susceptibility to infections, indicating a bidirectional relationship.^22,24^ Emerging evidence suggests that several viral and bacterial infections may impact the pathobiology of MAFLD, and those infections may take a more aggressive course in patients with MAFLD.^25^

There is a bidirectional relationship between the gut and liver, known as the gut–liver axis, wherein the gut microbiota and gut-derived metabolic byproducts directly affect the liver.^26^ On the other hand, liver-derived bile acids and antibodies directly affect the gut microbiota. Alterations in gut microbiota, increased production of proinflammatory cytokines, the integrity of the gut mucosal wall and gut-derived metabolic byproducts, including secondary bile acids, ethanol (produced by the ethanol-producing microbiota) and trimethylamine, contribute to the pathogenesis of MAFLD.^27^ Emerging evidence on the gut–liver axis and an understanding of the role played by intestinal microbiota in the pathobiology of MAFLD may assist with developing therapeutic targets against the disease.^28^

Another gap in the existing knowledge is the relationship between liver disease and skeletal muscle health. The muscle–liver–adipose tissue axis plays a major role in the pathogenesis and progression of MAFLD.^29^ Sarcopenia is characterized by a progressive loss of skeletal muscle mass, strength and function.^30^ Increased lipolysis mediated by insulin resistance generates free fatty acids, resulting in ectopic fat deposition in skeletal muscles (myosteatosis) and hepatic parenchyma (MAFLD).^31^ Insulin resistance also mediates increased proteolysis and muscle catabolism. A reduced lean muscle mass, together with myosteatosis, further exacerbates insulin resistance and mitigates the free fatty acid uptake by the skeletal muscle tissue, leading to worsening MAFLD.^32^ MAFLD, in turn, worsens sarcopenia through hyperammonemia, proinflammatory cytokine production and alterations in gut microbiota. Thus, there is a bidirectional relationship between MAFLD and sarcopenia.^32^

There is also a bidirectional association between OSA and MAFLD, with OSA being associated with a twofold increased risk of developing MAFLD, steatohepatitis and fibrosis, independent of obesity.^33^ The prevalence of MAFLD is 85% in patients with severe OSA, 26% of whom progress to hepatic fibrosis.^34,35^ This association was also observed in children: 44% of children with OSA who are not obese compared with 68% of children with OSA who are obese were found to have MAFLD.^36^ OSA-mediated chronic intermittent hypoxia resulting in insulin resistance, oxidative stress, alterations in gut microbiota and molecular changes contributes to the pathogenesis of MAFLD.^34^

MAFLD and polycystic ovary syndrome (PCOS) share various common risk factors, including central obesity, insulin resistance, chronic low-grade inflammation and hyperandrogenaemia; of these, insulin resistance plays the major role in the pathogenesis of both MAFLD and PCOS.^37^ Therefore, the prevalence of type 2 diabetes mellitus is also higher among these patients.^38^ Hyperandrogenaemia suppresses the low-density lipoprotein receptor gene transcription, prolonging the half-l ife of very low-density lipoprotein and low-density lipoprotein, resulting in hepatic lipid accumulation.^39^ Moreover, hyperandrogenaemia stimulates the transcription of androgen receptors and increases the release of tumour necrosis factor α. This causes chronic low-grade inflammation, which is involved in the pathogenesis of MAFLD.^40^

Increased rates of adverse pregnancy outcomes, including gestational diabetes mellitus, hypertension and caesarean delivery, are reported in pregnant women with MAFLD compared with pregnant women with NAFLD without metabolic dysfunction.^41^ The chronic low-grade inflammation associated with obesity, insulin resistance and MAFLD are the likely mechanisms for adverse maternal outcomes.^42,43^ Offspring borne to women with MAFLD may be more likely to exhibit early onset of adulthood obesity and MAFLD.^44^ Moreover, offspring who received fewer than 6 months of exclusive breastfeeding are found to have a significantly higher risk of developing MAFLD and an adverse metabolic profile in late adolescence.^43,45^

Liver biopsy is the gold-standard test used to diagnose MAFLD.^46^ Noninvasive investigations of MAFLD include biomarkers such as the NAFLD fibrosis score, the fibrosis-index 4, the enhanced liver fibrosis test and imaging modalities such as conventional ultrasound, velocity-controlled transient elastography (also known as FibroScan®; Echosens, Paris, France), acoustic radiation force impulse elastography, magnetic resonance elastography and computed tomography.^47^ Patients with MAFLD should be assessed for various extrahepatic complications associated with the disease, including chronic kidney disease, breast cancer, colorectal cancer, PCOS, osteoporosis, and cardiac arrhythmias.^5^

A loss of at least 3–5% of baseline body weight is needed to improve hepatic steatosis; however, a greater weight loss (up to 10%) may be needed to improve steatohepatitis and fibrosis.^48^ No drugs have been approved by the United States Food and Drug Administration for MAFLD at this stage; nonetheless, a few drugs, including obeticholic acid, elafibranor and selonsertib, have progressed to phase III development, showing evidence of reducing NASH and fibrosis.^49–52^ Several antidiabetic and antiobesity medications can be effectively used in patients with MAFLD, especially when patients have these comorbidities.^47^

**Figure 2:**
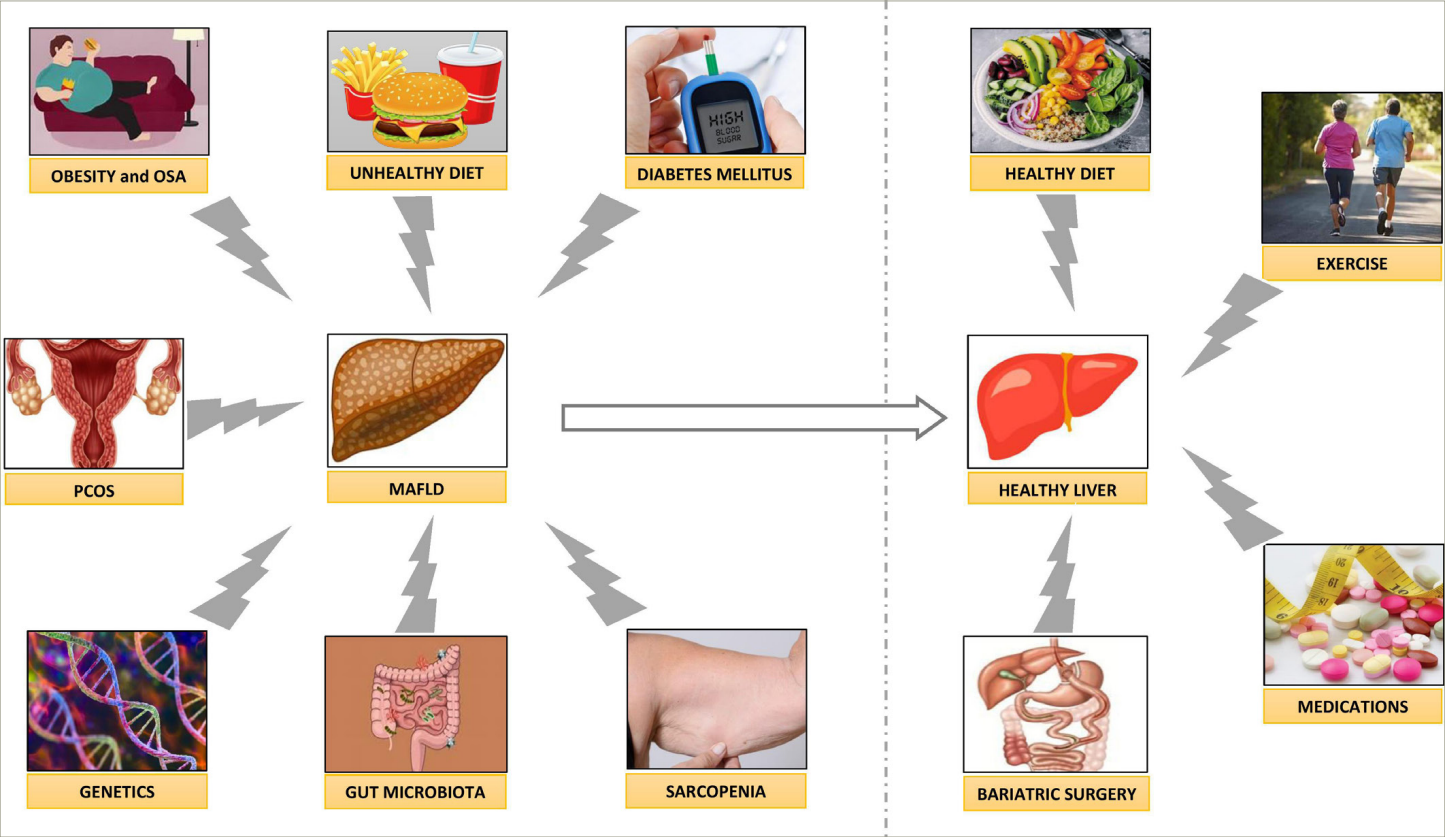
A schematic diagram showing the risk factors for development of metabolic-associated fatty liver disease and its management principles

*MAFLD = metabolic-associated fatty liver disease; OSA = obstructive sleep apnoea; PCOS = polycystic ovary syndrome*.

Epidemiological studies and Mendelian randomization studies have shown NAFLD/MAFLD to be a significant cardiovascular disease (CVD) risk factor, similarly to the CVD risk associated with diabetes mellitus.^53^ Hence, patients with NAFLD/MAFLD should undergo screening for CVD risk factors. The CVD risk associated with NAFLD/MAFLD can be reduced by intense lifestyle changes, pharmacological interventions (including glucagon-l ike peptide 1 receptor agonist, orlistat and naltrexone/ bupropion) or weight reduction via bariatric surgery, and intensive lipid-lowering and blood pressure-l owering therapy.^54^

Although MAFLD is a very common disease, affecting more than one-third of the global population, there are still knowledge gaps in the genetic, pathobiological and therapeutic aspects of the disease. Intense lifestyle interventions are the cornerstones of disease management. There are not any currently approved pharmacotherapeutic agents to cure this disease. A potential therapeutic algorithm for managing MAFLD based on current evidence is shown in *Figure 2*. The journal issue edited by Fouda and Pappachan, ‘Metabolic-associated Fatty Liver Disease', is a good attempt of updating the best evidence on this enigmatic disease.^2^ More evidence from on-going research is expected to enhance the global efforts to accelerate the actions against this devastating pandemic, which threatens human health and well-being.
